# Correlation between iron deposition and cognitive function in mild to moderate Alzheimer’s disease based on quantitative susceptibility mapping

**DOI:** 10.3389/fnagi.2024.1485530

**Published:** 2024-10-16

**Authors:** Yuqi Zhi, Ting Huang, Shanwen Liu, Meng Li, Hua Hu, Xiaoyun Liang, Zhen Jiang, Jiangtao Zhu, Rong Liu

**Affiliations:** ^1^Department of Radiology, The Second Affiliated Hospital of Soochow University, Suzhou, China; ^2^Department of Neurology, The Second Affiliated Hospital of Soochow University, Suzhou, China; ^3^Institute of Artificial Intelligence and Clinical Innovation, Neusoft Medical Systems Co., Ltd., Shanghai, China; ^4^Florey Institute of Neuroscience and Mental Health, The University of Melbourne, Melbourne, VIC, Australia

**Keywords:** Alzheimer’s disease, quantitative susceptibility mapping, iron, cognitive function, magnetic susceptibility values

## Abstract

**Background:**

Alzheimer’s disease (AD) is a complex neurodegenerative disorder characterized by progressively worsening cognitive decline and memory loss. Excessive iron accumulation produces severe cognitive impairment. However, there are no uniform conclusions about changes in brain iron content in AD. This study aimed to investigate the iron content of the deep brain nuclei in AD, and its correlation with cognitive function.

**Methods:**

Thirty-one patients with mild to moderate AD, 17 patients with mild cognitive impairment (MCI), and 20 age-, sex-, and education-matched healthy controls (HC) were collected. The QSM was used to quantify the magnetic susceptibility values of the caudate nucleus, putamen, globus pallidus, substantia nigra, red nucleus, and dentate nucleus, and to analyze the differences that existed between the three groups. As well as the correlation between the magnetic susceptibility values and cognitive function was calculated.

**Results:**

The magnetic susceptibility values of bilateral globus pallidus, left putamen, and bilateral substantia nigra were significantly higher in AD patients than in HC, and the magnetic susceptibility values of the right globus pallidus were significantly higher in AD patients than in MCI (all *p* < 0.05). The magnetic susceptibility values of the left dentate nucleus in the AD group were negatively correlated with the writing function of the MMSE subitem (*r* = −0.42, *p* = 0.020), and the magnetic susceptibility values of the left caudate nucleus and right dentate nucleus were significantly and negatively correlated with the naming function and language function of the MoCA subitem, respectively (*r* = −0.43, *p* = 0.019; *r* = −0.36, *p* = 0.048).

**Conclusion:**

Magnetic susceptibility values based on QSM correlate with cognitive function are valuable in discriminating AD from MCI and AD from HC.

## 1 Introduction

Alzheimer’s disease (AD) is a complex neurodegenerative disorder characterized by progressively worsening cognitive decline (Albertini et al., 2012) and memory loss (Jahn, 2013). Although the etiology of AD is not fully understood, clinical and neuropathological studies have identified β-amyloid (Aβ) plaques and tau neurofibrillary tangles as the main histopathological hallmarks of AD (Sisodia and Price, 1995; Hampel et al., 2021). With the deepening of research on the etiology of AD, experiments based on animal models have found that excessive iron accumulation due to an imbalance in iron metabolism produces severe cognitive impairment (Schröder et al., 2013). In addition to this, alterations in iron metabolism have been confirmed to be associated with Aβ plaques and tau neurofibrillary tangles according to histochemical, histopathological, and imaging studies (Everett et al., 2014; Gong et al., 2019; O’Callaghan et al., 2017). Iron is an important metal ion for the maintenance of many basic biological activities, including oxygen transport, DNA synthesis, mitochondrial respiration, myelin synthesis, and neurotransmitter synthesis and metabolism (Wang and Pantopoulos, 2011). Iron is retained in neural tissues primarily as ferritin or hemosiderin, and iron equilibrium is critical for maintaining normal brain function (Du et al., 2018).

QSM is a non-invasive MRI technique that can be used to quantify the distribution of magnetic susceptibility *in vivo*, and post-mortem validation studies have shown a strong correlation between iron content in grey matter and magnetic susceptibility values (Langkammer et al., 2012). The QSM technique has been utilized in several research to investigate changes in brain iron distribution and levels in normal and pathological conditions (Li et al., 2022; Chen et al., 2021; Sun et al., 2020). QSM is expected to provide imaging biomarkers to help with the diagnosis of AD. In this study, we used QSM to analyze the differences in magnetic susceptibility values of deep brain nucleus in AD, MCI, and HC. Additionally, to better understand the relationship between various aspects of cognitive function and iron deposition, a correlation study between cognitive function scores, including sub-items, and magnetic susceptibility values was conducted.

## 2 Materials and methods

### 2.1 Participants

This study included 31 mild-to-moderate AD patients and 17 MCI patients diagnosed from November 2022 to December 2023, as well as 20 age-, gender-, education-matched healthy controls recruited from local communities. The study was approved by the Ethics Committee of the hospital, and the subjects signed an informed consent form.

Inclusion criteria: (1) Meet the core diagnostic criteria for what is likely to be AD dementia, which were jointly developed by the National Institute on Aging (NIA) and the Alzheimer’s Association (AA) in 2011 (McKhann et al., 2011). (2) The diagnostic criteria for MCI also refer to the NIA-AA Recommended Guidelines: normal activities of daily living, subjective and objective cognitive impairment, but not yet dementia criteria (Albert et al., 2011). (3) Age 50–85 years, right-handed. Able to read and understand the content of the research scale.

Exclusion criteria: (1) Other diseases causing cognitive impairment, such as cerebrovascular disease, brain tumor, Hachinski ischemia score ≥4. (2) Severe medical diseases, such as cardiopulmonary, hepatic and renal insufficiency, hypothyroidism, malignant tumors and other chronic wasting diseases. (3) Other psychiatric diseases such as severe depression and schizophrenia. (4) Those who have incomplete clinical data and cannot cooperate with the cognitive function examination. (5) Those who have contraindications to magnetic resonance examination.

### 2.2 Neuropsychometric measures

The researchers received specific coherence training. Participants underwent neuropsychological testing in a quiet room. Cognitive function was tested using the Mini-Mental State Examination (MMSE) and the Montreal Cognitive Assessment (MoCA). The MMSE scale consisted of 11 sub-items: temporal orientation, spatial orientation, transient memory, delayed memory, numeracy, naming, retelling, executive functioning, reading, writing, and drawing. The MoCA scale consists of 7 sub-items: visuospatial and executive function, naming, attention, language, abstraction, delayed memory, and orientation.

### 2.3 MRI acquisition and processing

All subjects underwent cranial magnetic resonance scans on a 3.0 T MRI scanner (Prisma, Siemens Healthcare, Erlangen, Germany) equipped with a 64-channel phased array head coil. T1-weighted (T1w) imaging was performed using a three-dimensional magnetization prepared rapid acquisition gradient recalled echo (3D MP-RAGE), sequence with parameters: TR/TE = 2,300/3 ms, TI = 900 ms, spatial resolution = 0.8 × 0.8 × 0.8 mm^3^. A 3D gradient echo (3D-GRE) sequence was employed for QSM, consisted of six echoes with acquisition parameters: TR = 46.0 ms, TE = 7.25, 13.93, 20.61, 27.29, 33.97, 40.65 ms, flip angle = 15°, FOV = 220 mm × 220 mm, voxel size = 0.6 mm × 0.6 mm × 2.0 mm, slice thickness = 2.0 mm, scan time = 9: 01 min: s. To obtain the QSM maps, the 3D-GRE was processed using the MEDI toolbox, a post-processing package that runs on MATLAB (Ver. 2016b, Matrix Laboratory, MathWorks).

### 2.4 Selection of region of interest

**Figure 1:**
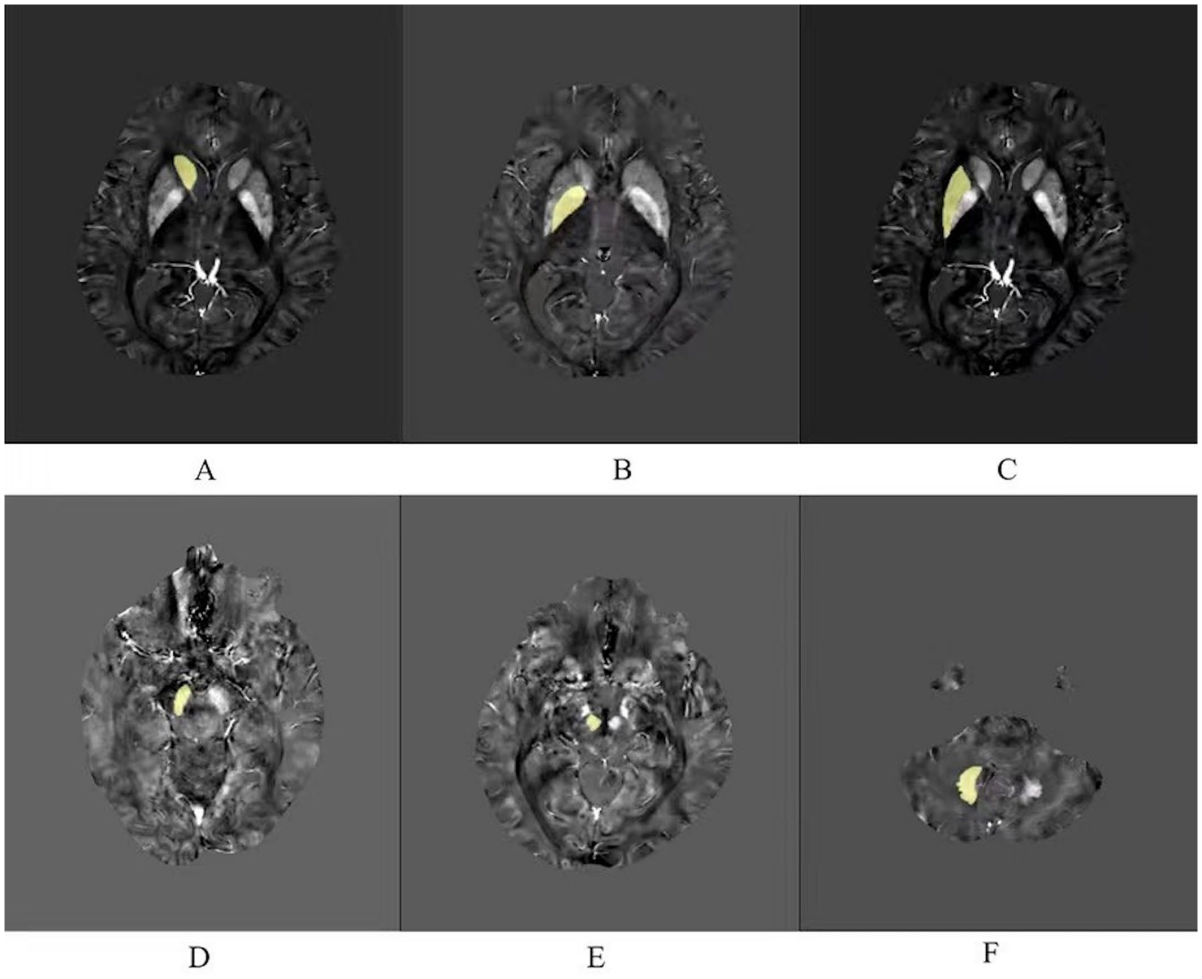
Six regions of interest (ROIs) analyzed in this study. (A) Caudate nucleus. (B) Globus pallidus. (C) Putamen. (D) Substantia nigra. (E) Red nucleus. (F) Dentate nucleus.

As shown in the Figure 1, iron-rich deep brain nuclei were selected (Haacke et al., 2005), including the bilateral caudate nucleus (Cd), globus pallidus (Gp), putamen (Pt), substantia nigra (Sn), red nucleus (Rn), and dentate nucleus (Dn). Manual outlining was performed using MRIcroN by two physicians after unified training. Images were magnified 6-fold during outlining to ensure clear nucleus edges; voxels at the nucleus boundaries were excluded from the region of interest (ROI) to minimize partial volume effects.

### 2.5 Statistical analysis

Statistical analyses were performed using SPSS 26.0 and MedCalc 20.0 software packages. Gender variables were expressed as percentages and comparisons between groups were performed using the *χ2* test. Normally distributed measures were expressed as mean ± standard deviation, non-normally distributed data were expressed as Μ(P_25_, P_75_). One-way ANOVA was used to compare data that conformed to normal distribution and homogeneity of variance, otherwise the Kruskal–Wallis *H* test was used, and the Bonferroni test was used for *post hoc* comparisons. Comparison of disease duration between AD and MCI was performed using the Mann–Whitney *U* test. The correlation between magnetic susceptibility values and cognitive function scores was analyzed using partial correlation, controlling for the age variable. The diagnostic efficacy of magnetic susceptibility values was assessed using the receiver operating characteristic curve (ROC) and area under curve (AUC). The consistency of the ROI results outlined by the two physicians was assessed using the intraclass correlation coefficient (ICC), with an ICC value greater than 0.75 as high consistency, between 0.40 and 0.75 as good consistency, and less than 0.4 as poor consistency.

## 3 Result

### 3.1 Comparison of clinical data of participants

As shown in the Table 1, there were no statistical differences in gender, age, and years of education among the three groups (*χ*^2^ = 0.98, *p* = 0.611; *F* = 1.40, *p* = 0.255; *H* = 1.85; *p* = 0.397, respectively). There was no statistical difference in disease duration between AD and MCI (*Z* = −1.247, *p* = 0.213). MMSE and MoCA scores differed statistically between the three groups (*H* = 50.97, *p* < 0.001; *H* = 52.03, *p* < 0.01, respectively).

**Table 1:** Demographic characteristics and cognitive scores of study subjects.

Characteristic	AD (*n* = 31)	MCI (*n* = 17)	HC (*n* = 20)	Statistics	*p*-value
Male (*n*, %)	9 (29.0%)	6 (41.2%)	8 (40.0%)	*χ*^2^ = 0.98	0.611
Female (*n*, %)	22 (71.0%)	11 (58.8%)	12 (60.0%)		
Age	71.5 ± 9.8	70.8 ± 7.8	67.3 ± 7.4	*F* = 1.40	0.255
Years of education	9 (2, 12)	8.6 ± 4.6	10.6 ± 4.4	*H* = 1.85	0.397
Disease duration (months)	24 (18, 36)	24 (8, 36)	NA	*Z* = -1.25	0.213
MMSE	20 ± 4	27 ± 2	28 (27, 30)	*H* = 50.97	<0.001^ab***^
Temporal orientation	3 (1, 3)	4 (4, 5)	5 (5, 5)	*H* = 41.24	<0.01^ab**^
Spatial orientation	4 (3, 4)	5 (4, 5)	5 (5, 5)	*H* = 31.46	<0.001^ab***^
Transient memory	3 (3, 3)	3 (3, 3)	3 (3, 3)	*H* = 3.12	0.210
Delayed memory	0 (0, 1)	2 (1, 3)	3 (2, 3)	*H* = 32.23	<0.01^ab**^
Numeracy	4 (3, 5)	5 (4, 5)	5 (5, 5)	*H* = 9.99	<0.05^b*^
Naming	2 (2, 2)	2 (2, 2)	2 (2, 2)	*H* = 0.79	0.673
Retelling	1 (1, 1)	1 (1, 1)	1 (1, 1)	*H* = 1.75	0.417
Executive functioning	2 (2, 3)	3 (2, 3)	3 (3, 3)	*H* = 9.88	<0.01^b**^
Reading	1 (1, 1)	1 (1, 1)	1 (1, 1)	*H* = 3.69	0.158
Writing	1 (0, 1)	1 (1, 1)	1 (1, 1)	*H* = 8.25	<0.05^b*^
Drawing	1 (0, 1)	1 (1, 1)	1 (1, 1)	*H* = 2.41	0.299
MoCA	16 ± 4	22 ± 3	27 (26, 27)	*H* = 52.03	<0.01
Visuospatial and executive function	2 (1, 4)	4 (3, 5)	5 (4, 5)	*H* = 21.04	<0.001^b***^
Naming	2 (1, 3)	3 (2, 3)	3 (2, 3)	*H* = 10.42	<0.01^b**^
Attention	6 (4, 6)	6 (6, 6)	6 (5, 6)	*H* = 3.66	0.160
Language	1 (0, 2)	2 (1, 2)	2 (2, 3)	*H* = 14.42	<0.01^b**^
Abstraction	1 (0, 2)	2 (1, 2)	2 (1, 2)	*H* = 14.05	<0.001^b***^
Delayed memory	0 (0, 0)	0 (0, 3)	4 (3, 5)	*H* = 46.34	<0.001^bc***^
Orientation	3 (3, 4)	6 (5, 6)	6 (6, 6)	*H* = 39.39	<0.001^ab***^

AD, Alzheimer’s disease; MCI, mild cognitive impairment; HC, health control; MMSE, Mini-Mental State Examination; MoCA, Montreal Cognitive Assessment; NA, not applicable. Data are presented as mean ± SD, M(P_25_, P_75_), or counts (percentages). ^a^For the comparison between AD and MCI groups. ^b^For the comparison between AD and HC groups. ^c^For the comparison between HC and MCI groups. ^*^p < 0.05, ^**^p < 0.01, and ^***^p < 0.001.

### 3.2 Comparison of magnetic susceptibility values of ROIs between the three groups

The ICC values of the magnetic susceptibility values for each nucleus were all greater than 0.75, with high intra-group consistency (Table 2), and the mean of the magnetic susceptibility values obtained by the two physicians was used for statistical analysis (Table 3). Statistical differences in the magnetic susceptibility values of the bilateral globus pallidus and the substantia nigra, and the left putamen were found between the three groups (all *p* < 0.05). *Post hoc* analysis of the above nuclei showed that the magnetic susceptibility values of the bilateral globus pallidus and the substantia nigra, and the left putamen were significantly higher in AD than in HC (all *p* < 0.05). The magnetic susceptibility values of the right globus pallidus were significantly higher in AD than in MCI (*p* < 0.05).

**Table 2:** The ICC values of the magnetic susceptibility values for ROIs.

ROIs	AD		MCI		HC	
	ICC	95% CI	ICC	95% CI	ICC	95% CI
L-Cd	0.97	0.938–0.985	0.921	0.796–0.971	0.851	0.661–0.938
R-Cd	0.975	0.949–0.988	0.938	0.839–0.977	0.837	0.634–0.932
L-Gp	0.968	0.934–0.984	0.983	0.955–0.994	0.829	0.617–0.928
R-Gp	0.973	0.944–0.987	0.963	0.900–0.986	0.929	0.830–0.971
L-Pt	0.966	0.931–0.983	0.983	0.953–0.994	0.893	0.750–0.956
R-Pt	0.979	0.957–0.990	0.975	0.933–0.991	0.911	0.790–0.964
L-Sn	0.981	0.961–0.991	0.988	0.968–0.996	0.948	0.873–0.979
R-Sn	0.991	0.982–0.996	0.986	0.962–0.995	0.948	0.873–0.979
L-Rd	0.968	0.935–0.985	0.986	0.962–0.995	0.956	0.892–0.982
R-Rd	0.968	0.935–0.985	0.973	0.928–0.990	0.954	0.887–0.981
L-Dn	0.969	0.936–0.985	0.987	0.965–0.995	0.966	0.917–0.987
R-Dn	0.965	0.928–0.983	0.982	0.951–0.993	0.941	0.858–0.976

AD, Alzheimer’s disease; MCI, mild cognitive impairment; HC, health control; L, left; R, right; Cd, caudate nucleus; Gp, globus pallidus; Pt, putamen; Sn, substantia nigra; Rn, red nucleus; Dn, dentate nucleus.

**Table 3:** Comparison of magnetic susceptibility values of ROIs between the three groups.

ROIs	AD	MCI	HC	Statistics	*p*-value
L-Cd	84.670 ± 19.602	79.434 ± 19.867	76.535 ± 17.206	*F* = 1.19	0.311
R-Cd	88.121 ± 24.035	76.094 ± 20.948	74.229 ± 19.918	*F* = 2.95	0.059
L-Pt	90.401 ± 28.041	86.823 ± 36.968	67.349 ± 18.887	*H* = 9.03	<0.01^b**^
R-Pt	89.134 ± 32.391	85.880 ± 36.212	71.311 ± 20.531	*H* = 4.67	0.102
L-Gp	158.230 ± 29.764	145.255 ± 37.804	132.677 ± 27.312	*F* = 4.11	<0.05^b*^
R-Gp	162.817 ± 32.919	139.785 ± 31.638	131.152 ± 24.342	*F* = 7.41	<0.05^ab*^
L-Sn	157.449 ± 40.222	125.757 ± 46.247	107.018 ± 24.497	*H* = 16.85	<0.001^b***^
R-Sn	161.160 ± 43.839	135.749 ± 44.211	117.311 ± 20.395	*H* = 14.20	<0.01^b**^
L-Rn	118.338 ± 27.282	126.293 ± 45.389	108.394 ± 25.386	*H* = 2.09	0.234
R-Rn	124.875 ± 29.870	132.785 ± 39.216	118.579 ± 27.963	*F* = 0.91	0.407
L-Dn	103.748 ± 29.947	114.779 ± 40.630	98.404 ± 30.445	*F* = 1.17	0.318
R-Dn	106.013 ± 28.182	121.062 ± 34.756	96.319 ± 29.857	*F* = 3.07	0.053

AD, Alzheimer’s disease; MCI, mild cognitive impairment; HC, health control; ROI, region of interest; L, left; R, right; Cd, caudate nucleus; Gp, globus pallidus; Pt, putamen; Sn, substantia nigra; Rn, red nucleus; Dn, dentate nucleus. Magnetic susceptibility values are expressed using mean ± SD in part per billion (ppb). The results of statistically significant differences are indicated with ^a^for the comparison between AD and MCI groups, ^b^for the comparison between AD and HC groups, ^c^for the comparison between MCI and HC groups. The significance for data is Bonferroni corrected *p* = 0.05, ^*^p < 0.05, ^**^p < 0.01, and ^***^p < 0.001.

### 3.3 ROC curve analysis of magnetic susceptibility values for ROIs that differed between the three groups

**Figure 2:**
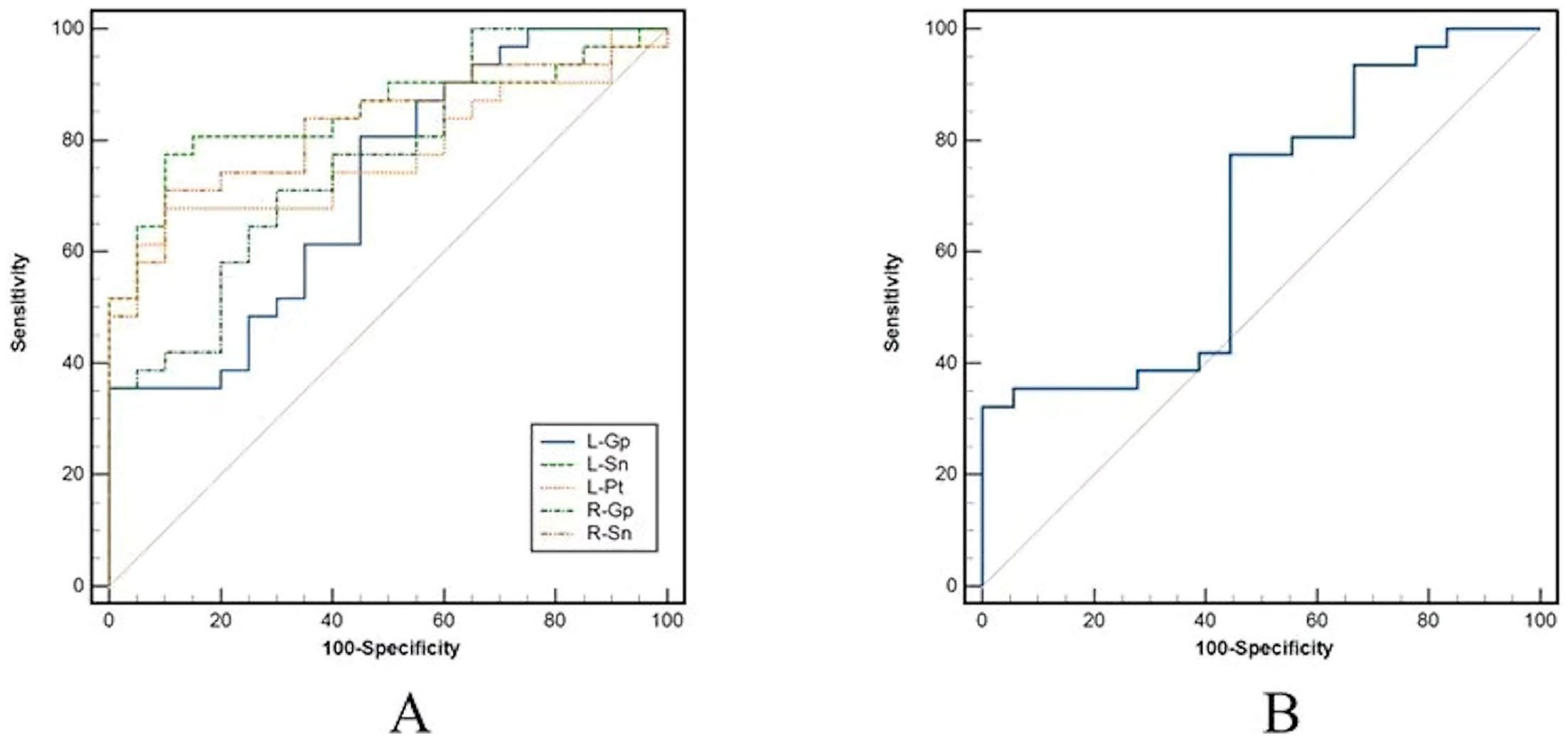
Receiver operator characteristic (ROC) curves analysis for magnetic susceptibility values. (A) Results of ROC curve analysis for ROIs with differences between AD and HC. (B) Results of ROC curve analysis for right pallidum with differences between AD and MCI. AD, Alzheimer’s disease; MCI, mild cognitive impairment; HC, health control; ROI, regions of interest.

Magnetic susceptibility values based on ROIs with significant differences identified AD and HC, left putamen (AUC = 0.776, *p* < 0.001), left globus pallidus (AUC = 0.716, *p* = 0.004), right globus pallidus (AUC = 0.763, *p* < 0.001), left substantia nigra (AUC = 0.848, *p* < 0.001), right substantia nigra (AUC = 0.826, *p* < 0.001); to identify AD and MCI, right globus pallidus (AUC = 0.663, *p* = 0.049) (Table 4 and Figure 2).

**Table 4:** ROC curve analysis of magnetic susceptibility values for ROIs that differed between the groups.

ROIs	Groups	SE (%)	SP (%)	AUC	Criterion	*p*-value
L-Pt	AD vs. HC	67.7	90.0	0.776	>85.055	<0.001
L-Gp	AD vs. HC	80.6	55.0	0.716	>131.143	0.004
R-Gp	AD vs. HC	71.0	70.0	0.763	>141.328	<0.001
	AD vs. MCI	77.4	55.6	0.663	>136.264	0.049
L-Sn	AD vs. HC	77.4	90.0	0.848	>133.893	<0.001
R-Sn	AD vs. HC	71.0	90.0	0.826	>139.897	<0.001

AD, Alzheimer’s disease; MCI, mild cognitive impairment; HC, health control; ROC, receiver operating characteristic; AUC, area under the ROC curve; SE, sensitivity; SP, specificity; L, left; R, right; Gp, globus pallidus; Pt, putamen; Sn, substantia nigra.

### 3.4 Partial correlation analysis between magnetic susceptibility values of ROIs and cognitive function

Using age as a control variable, magnetic susceptibility values in the left dentate nucleus of the AD were significantly negatively correlated with the sub-item writing function of the MMSE (*r* = −0.42, *p* = 0.020), and in the left caudate nucleus and the right dentate nucleus were significantly negatively correlated with the sub-item naming function of the MoCA, and language function, respectively (*r* = −0.43, *p* = 0.019; *r* = −0.36, *p* = 0.048) (Tables 5, 6).

**Table 5:** Partial correlation analysis between magnetic susceptibility values of ROIs and MMSE scores in AD group.

MMSE and its sub items		Cd		Gp		Pt		Sn		Rn		Dn	
		*ρ*	*p*	*ρ*	*p*	*ρ*	*p*	*ρ*	*p*	*ρ*	*p*	*ρ*	*p*
MMSE	L	0.03	0.871	−0.05	0.788	0.06	0.766	−0.15	0.425	0.09	0.655	−0.01	0.943
	R	0.12	0.534	−0.02	0.907	0.016	0.396	0.04	0.835	0.10	0.604	0.13	0.508
Temporal orientation	L	0.00	0.991	0.00	0.993	0.07	0.713	0.11	0.567	0.11	0.554	0.28	0.138
	R	0.14	0.462	0.00	0.991	0.05	0.789	0.12	0.535	0.28	0.135	0.31	0.092
Spatial orientation	L	0.06	0.754	−0.21	0.259	0.16	0.408	−0.19	0.306	−0.01	0.947	−0.19	0.320
	R	0.00	0.984	−0.28	0.140	0.09	0.623	−0.01	0.948	0.08	0.684	−0.11	0.573
Transient memory	L	0.27	0.151	−0.07	0.731	0.14	0.453	0.01	0.945	−0.19	0.308	0.05	0.808
	R	0.29	0.119	0.04	0.856	0.28	0.139	0.06	0.757	−0.12	0.519	0.12	0.540
Delayed memory	L	−0.31	0.095	0.00	0.995	−0.13	0.497	0.31	0.101	0.12	0.546	0.36	0.051
	R	0.08	0.685	0.11	0.580	−0.23	0.214	0.26	0.160	0.29	0.120	0.22	0.248
Numeracy	L	0.05	0.803	0.05	0.811	0.09	0.655	0.12	0.521	0.16	0.398	−0.15	0.426
	R	−0.05	0.788	0.01	0.977	0.23	0.214	0.13	0.511	0.10	0.615	−0.02	0.902
Naming	L	0.08	0.666	−0.01	0.971	0.12	0.533	−0.29	0.116	−0.16	0.402	−0.04	0.852
	R	−0.13	0.480	−0.06	0.750	0.08	0.671	−0.33	0.071	−0.14	0.447	0.23	0.225
Retelling	L	0.04	0.847	0.16	0.389	0.13	0.503	−0.16	0.401	−0.10	0.618	0.02	0.938
	R	0.01	0.944	0.14	0.479	0.13	0.505	−0.07	0.711	−0.20	0.282	0.23	0.125
Executive functioning	L	0.05	0.785	0.05	0.792	0.13	0.500	−0.01	0.961	−0.14	0.477	−0.27	0.158
	R	0.02	0.913	0.00	0.985	0.06	0.746	−0.10	0.585	−0.23	0.225	−0.20	0.293
Reading	L	0.05	0.814	0.24	0.212	0.16	0.408	0.13	0.509	0.23	0.216	−0.07	0.704
	R	0.10	0.617	0.16	0.398	0.19	0.307	0.20	0.284	−0.01	0.942	−0.01	0.962
Writing	L	0.18	0.351	−0.05	0.795	0.22	0.235	−0.07	0.715	0.01	0.948	−0.42	0.020*
	R	0.16	0.388	−0.05	0.808	0.33	0.079	−0.01	0.971	−0.13	0.495	−0.26	0.164
Drawing	L	−0.04	0.852	−0.32	0.089	−0.03	0.888	−0.33	0.080	−0.04	0.842	−0.23	0.219
	R	−0.04	0.819	−0.25	0.175	0.09	0.641	−0.28	0.134	0.01	0.974	−0.15	0.416

AD, Alzheimer’s disease; ROI, region of interest; L, left; R, right; Cd, caudate nucleus; Gp, globus pallidus; Pt, putamen; Sn, substantia nigra; Rn, red nucleus; Dn, dentate nucleus; MMSE, Mini-Mental State Examination. *ρ*, Spearman’s correlation coefficient. ^*^p < 0.05.

**Table 6:** Partial correlation analysis between magnetic susceptibility values of ROIs and MoCA scores in AD group.

MMSE and its sub items		Cd		Gp		Pt		Sn		Rn		Dn	
		*ρ*	*p*	*ρ*	*p*	*ρ*	*p*	*ρ*	*p*	*ρ*	*p*	*ρ*	*p*
MoCA	L	−0.08	0.695	−0.16	0.405	0.03	0.869	−0.258	0.203	0.111	0.467	−0.08	0.698
	R	0.06	0.767	−0.09	0.643	0.20	0.293	−0.24	0.724	0.10	0.603	0.15	0.432
visuospatial and executive function	L	−0.03	0.868	−0.08	0.695	0.18	0.356	−0.07	0.379	0.08	0.682	−0.21	0.277
	R	0.04	0.825	−0.07	0.704	0.32	0.090	−0.02	0.901	0.08	0.665	0.06	0.772
Naming	L	−0.43^*^	0.019	0.01	0.953	−0.04	0.847	0.05	0.795	0.16	0.385	−0.15	0.415
	R	−0.30	0.102	0.10	0.603	0.00	0.988	0.09	0.652	0.15	0.423	−0.09	0.643
Attention	L	0.19	0.323	−0.04	0.830	0.04	0.829	−0.16	0.414	−0.01	0.951	−0.16	0.387
	R	0.01	0.947	−0.02	0.913	0.24	0.194	−0.06	0.773	−0.01	0.593	−0.07	0.704
Language	L	0.02	0.916	−0.06	0.742	−0.2	0.280	0.02	0.917	0.13	0.506	−0.29	0.122
	R	−0.08	0.693	−0.06	0.745	−0.02	0.905	0.03	0.897	0.18	0.354	−0.36^*^	0.048
Abstraction	L	0.20	0.296	−0.04	0.839	0.12	0.522	−0.17	0.371	0.01	0.959	−0.20	0.300
	R	0.29	0.115	−0.05	0.791	0.35	0.061	0.06	0.764	−0.08	0.692	0.10	0.599
Delayed memory	L	−0.32	0.082	−0.12	0.540	−0.12	0.527	0.05	0.815	−0.29	0.121	0.01	0.957
	R	−0.14	0.449	−0.10	0.591	−0.19	0.311	−0.10	0.596	−0.07	0.705	0.05	0.802
Orientation	L	0.00	0.994	−0.09	0.636	0.13	0.484	0.11	0.552	0.16	0.395	0.28	0.133
	R	0.24	0.200	−0.12	0.514	0.03	0.874	0.14	0.448	0.32	0.087	0.30	0.106

AD, Alzheimer’s disease; ROI, region of interest; L, left; R, right; Cd, caudate nucleus; Gp, globus pallidus; Pt, putamen; Sn, substantia nigra; Rn, red nucleus; Dn, dentate nucleus; MoCA, Montreal Cognitive Assessment. *ρ*, Spearman’s correlation coefficient. ^*^p < 0.05.

## 4 Discussion

In this study, by using the QSM technique to analyze the magnetic susceptibility values of deep grey matter nuclei in AD, MCI, and HC, it was found that the magnetic susceptibility values of the bilateral globus pallidus and the substantia nigra, the left putamen of AD were significantly higher than those of HC, and that the magnetic susceptibility values of the right globus pallidus of AD were higher than those of MCI, but no nuclei were found to be significantly different between MCI and HC. The caudate nucleus, globus pallidus, putamen, thalamus, and substantia nigra together form the basal ganglia and have been correlated with cognitive and motor functions (Graybiel, 2000), and the imbalance of iron homeostasis may be associated with corresponding dysfunction. β-amyloid (Aβ) plaques and tau neurofibrillary tangles are known pathological markers of AD (Sisodia and Price, 1995; Hampel et al., 2021), and it has been suggested that there is an interaction between the aggregation of these two and iron deposition (Galante et al., 2018; Liu et al., 2018), which together contribute to the development of AD and affect its cognitive function. However, it is unclear whether iron deposition is a pathological mechanism affecting the development of AD or merely a manifestation of the disease progression. MCI is clinically manifested by mild cognitive decline, but daily life is not significantly affected and has the potential to develop into AD (Petersen et al., 2001). In the present study, we found that the magnetic susceptibility values of the right globus pallidus of AD were higher than those of MCI, which could potentially be a marker to aid in the differentiation of AD from MCI. Consistent with the findings of Cogswell et al. (2021) and Kim et al. (2017), no significant difference was found in the present study when MCI was compared with HC, although the magnetic susceptibility values of all nuclei were higher in MCI than in HC. It is possible that the disease progression is not linear with pathological changes or iron deposition, which requires further study.

Iron levels in the brain are not uniformly distributed, with the brain regions with the highest iron levels located in the basal ganglia (Ramos et al., 2014). Several studies (Adisetiyo et al., 2012; Aquino et al., 2009; Xu et al., 2008) have found a positive correlation between iron deposition in the basal ganglia and age, which may be due to the fact that the basal ganglia have a higher metabolic rate and are more vulnerable to the decrease in metabolic rate that occurs with age (Vitanova et al., 2019), leading to more significant increase in iron deposition.

Controlling for age, the present study found that magnetic susceptibility values of the dentate nucleus in AD were negatively correlated with writing and language functions. The dentate nucleus is the largest deep nucleus of the cerebellum, and in addition to its involvement in motor functions (Bond et al., 2017), it is associated with executive functions including planning, working memory, and language functions (Jardri et al., 2007; Gitelman et al., 2005; Kim et al., 1994). Imbalances in iron deposition homeostasis may affect the function of the dentate nucleus, which may exhibit corresponding dysfunction. The basal ganglia, an important structure of the brain, is usually associated with motor control, but the different regions that comprise it have significant functional differences (Alexander et al., 1986). The caudate nucleus, anatomically considered part of the basal ganglia, is associated with language processing, and damage to it can lead to aphasia, including functions such as naming, spelling, and pronunciation of words (Brunner et al., 1982). In the present study, we found that the magnetic susceptibility values of the left caudate nucleus were negatively correlated with naming function, which may be due to the increased iron deposition that leads to impaired language processing, thus affecting the naming function in AD. Some previous papers (Uchida et al., 2020; Uchida et al., 2022b; Uchida et al., 2019) reported that cognitive impairment in neurodegenerative diseases, such as Alzheimer’s disease and Parkinson’s disease, is related to the susceptibility changes in the basal ganglia. Alterations in the basal ganglia can lead to the cognitive deficits observed in these conditions. These changes may involve a variety of neurobiological mechanisms, including neurotransmitter imbalances, dysfunction of neural circuits, and neuroinflammation, which can impair cognitive function. In our manuscript, we aim to build on these findings by examining how specific susceptibility changes in the basal ganglia correlate with cognitive impairment in our patient cohort. We believe our study provides valuable insights into the complex interactions between these neurodegenerative processes.

In this study, the diagnostic efficacy of the magnetic susceptibility values of ROI was assessed using ROC curves, and the magnetic susceptibility values of the left putamen, bilateral globus pallidus and substantia nigra had good diagnostic efficacy in discriminating between AD and HC (AUC >0.7), and average diagnostic efficacy in discriminating between AD and MCI using the magnetic susceptibility values of the right globus pallidus (AUC = 0.663 <0.7). This is an interesting finding. The AUC of 0.663 indicates a moderate ability to differentiate AD from MCI, suggesting that while the model shows promise, there may be significant overlap in susceptibility values between the two groups. This may be due to the fact that patients with mild to moderate AD were enrolled, whereas MCI is considered an early stage of AD, and there may be less variability in iron deposition between the two, or it may be due to the relationship between individual differences. This moderate efficacy underscores the need for further research to enhance the diagnostic accuracy, potentially through the integration of additional biomarkers or clinical parameters. A more nuanced understanding of these results could guide clinical decision-making and inform future studies.

The relationship between iron metabolism and the blood-brain barrier (BBB) is indeed critical in understanding neurodegenerative diseases like Alzheimer’s disease. Iron accumulation in the brain can exacerbate oxidative stress and neuroinflammation, processes that are pivotal in Alzheimer’s pathology. Previous studies (Uchida et al., 2022a) have shown that APOE ɛ4 dose is associated with increased brain iron levels and β-amyloid accumulation highlights a significant link between genetic risk factors and iron dysregulation. This suggests that individuals carrying the APOE ɛ4 allele may experience compromised BBB function, leading to altered iron transport and deposition in the brain, potentially accelerating neurodegenerative processes. Researchs (Uchida et al., 2023) on BBB imaging contributes to our understanding of the neurovascular unit’s pathophysiology in Alzheimer’s and related dementias. These imaging studies reveal how BBB dysfunction can facilitate the entry of iron and other potentially neurotoxic substances into the brain, further aggravating neuronal damage and cognitive decline. Together, these studies underscore the importance of the BBB in regulating iron homeostasis in the brain and its implications for neurodegenerative diseases. Understanding these mechanisms could pave the way for novel therapeutic strategies aimed at protecting the BBB and managing iron levels in the brain.

Several limitations of this study: firstly, the grouping regarding AD, MCI, and HC was based on scale scores rather than pathological findings such as cerebrospinal fluid biomarkers, but we have excluded dementia due to other neuropsychiatric disorders as much as possible. Secondly, the relatively small sample size of this study and the inclusion of patients with mild-to-moderate AD, which did not include severe AD, may have led to some limitations in the results. Thirdly, the ROIs selected for this study were only known iron-rich nuclei, which is a small study to comprehensively assess the correlation between brain iron content and cognitive function. Fourth, the present study is a cross-sectional design, which cannot dynamically assess the changes over time. These limitations need to be further refined in subsequent studies.

## 5 Conclusion

Magnetic susceptibility values based on QSM correlate with cognitive function are valuable in discriminating AD from MCI and AD from HC.

## Data Availability

The original contributions presented in the study are included in the article/supplementary material, further inquiries can be directed to the corresponding authors.
